# Influence of upper and temporal transconjunctival sclerocorneal incision on marginal reflex distance after cataract surgery

**DOI:** 10.1186/s12886-016-0286-1

**Published:** 2016-07-07

**Authors:** Rikiya Tamaki, Masahiko Gosho, Kyoichi Mizumoto, Nahoko Kato, Masahiro Zako

**Affiliations:** Department of Ophthalmology, Aichi Medical University, Nagakute, 480-1195 Aichi Japan; Department of Clinical Trial and Clinical Epidemiology, Faculty of Medicine, University of Tsukuba, Tsukuba, 305-8575 Ibaraki Japan

**Keywords:** Cataract, Marginal reflex distance, Ptosis, Transconjunctival sclerocorneal incision

## Abstract

**Background:**

Ptosis incidence following cataract surgery is reduced with a recently developed phacoemulsification technique using a small incision. However, it remains uncertain whether an upper transconjunctival sclerocorneal incision can cause minor blepharoptosis. In the present prospective study, patients underwent cataract surgery with either an upper or temporal 2.4-mm transconjunctival sclerocorneal incision. We measured the marginal reflex distance 1 (MRD1) preoperatively and postoperatively, and compared these measurements between the two different incision types. Further we explored the risk factors of the postoperative MRD1 reduction.

**Methods:**

The study population included patients who underwent cataract surgery on both eyes at Aichi Medical University between October 2013 and September 2015. In each patient, one eye was operated using an upper 2.4-mm transconjunctival sclerocorneal incision, and the other with a temporal incision. We prespecified that an MRD1 difference of ≥0.5 mm between the pre- and post-surgical measurements indicated postoperative ptosis, which was a strict criterion. MRD1 was measured using digital photography, and we calculated the difference between the preoperative and postoperative MRD1 values. This change in MRD1 was compared between the groups with different incision locations. The change in MRD1 was analyzed by using the multivariate regression model including incision position (temporal or upper), preoperative MRD1, and preoperative distance between medial and lateral canthi.

**Results:**

We assessed data from a total of 34 patients. The mean change in MRD1 from pre-operation to post-operation measurements was −0.26 ± 0.93 with the temporal incision and −0.24 ± 0.86 with the upper incision. The mean difference in the change in MRD1 between the different two incision types was −0.02, with a 95 % CI of −0.24 to 0.20, establishing equivalence between these incision types. The multivariate regression analysis showed that the preoperative MRD1 was significantly associated with the reduction of MRD1 after surgery (*p* = 0.034).

**Conclusions:**

Cataract surgery using upper and temporal 2.4-mm transconjunctival sclerocorneal incisions are clinically equivalent with regards to change in MRD1, and neither incision type caused critical postoperative ptosis. The longer preoperative MRD1 was significantly associated with the reduction of MRD1 after surgery.

**Trial registration:**

Current Controlled Trials UMIN000022310. Retrospectively registered 14 May 2016.

**Electronic supplementary material:**

The online version of this article (doi:10.1186/s12886-016-0286-1) contains supplementary material, which is available to authorized users.

## Background

Ptosis incidence following cataract surgery has been reduced with the use of a recently developed phacoemulsification technique involving a small incision, with reported rates of 4–21 % [1–8]. Ptosis is generally defined as a decrease in the marginal reflex distance 1 (MRD1) of 2 mm or more in the postoperative measurement compared to the preoperative measurement [9, 10]. The precise etiology of ptosis remains elusive, but is considered to be multifactorial. The most critical factor in postoperative ptosis appeared trauma to the superior rectus/levator complex caused by local anesthesia, superior rectus bridle suture, and lid speculum [1–7]. Preoperative ptosis showed no effect on postoperative ptosis [1], but preoperative visible iris sign was shown as a clinical sign of severe involutional ptosis [10]. Puvanachandra et al. reported the incidence of postoperative ptosis has reduced by changing from ECCE (18 %) to phacoemulsification (0 %) [9]. It remains uncertain whether the use of an upper transconjunctival sclerocorneal small incision to perform phacoemulsification in cataract surgery can lead to minor blepharoptosis after surgery, and no prior prospective study has addressed this question.

In the present study, patients underwent cataract surgery in both eyes, with an upper incision used in one eye and a temporal incision used in the other eye. We measured the MRD1 in each eye before and three months after phacoemulsification, and statistically analyzed the MRD1 values, using a strict criterion to define postoperative ptosis.

## Methods

### Patients

This study included patients who were scheduled for cataract surgery on both eyes at Aichi Medical University between October 2013 and September 2015. Patients were excluded if they had a history of thyroid eye disease, proptosis, enophthalmos, or previous lid or ocular surgery.

### Cataract surgery

All phacoemulsification surgeries were performed by one surgeon (R.T.). Surgery was performed with topical and intracameral local anesthesia. Since evidence suggests that a metallic persistent eyelid speculum may lead to postoperative ptosis [11], here we used a disposable flexible EzSpec lid speculum (Hoya, Tokyo, Japan) for all patients. No superior rectus bridle suture was used. A 2.4-mm sutureless upper or temporal transconjunctival sclerocorneal incision was performed. The patients were randomly prospectively assigned to either Group 1 (upper incision for right eye and temporal incision for left eye) or Group 2 (upper incision for left eye and temporal incision for right eye), such that each patient received one of each incision type. Another 0.8-mm clear corneal incision was made at the nasal side in all patients.

### Measurement of MRD1 and distance between medial and lateral canthi

**Fig. 1:**
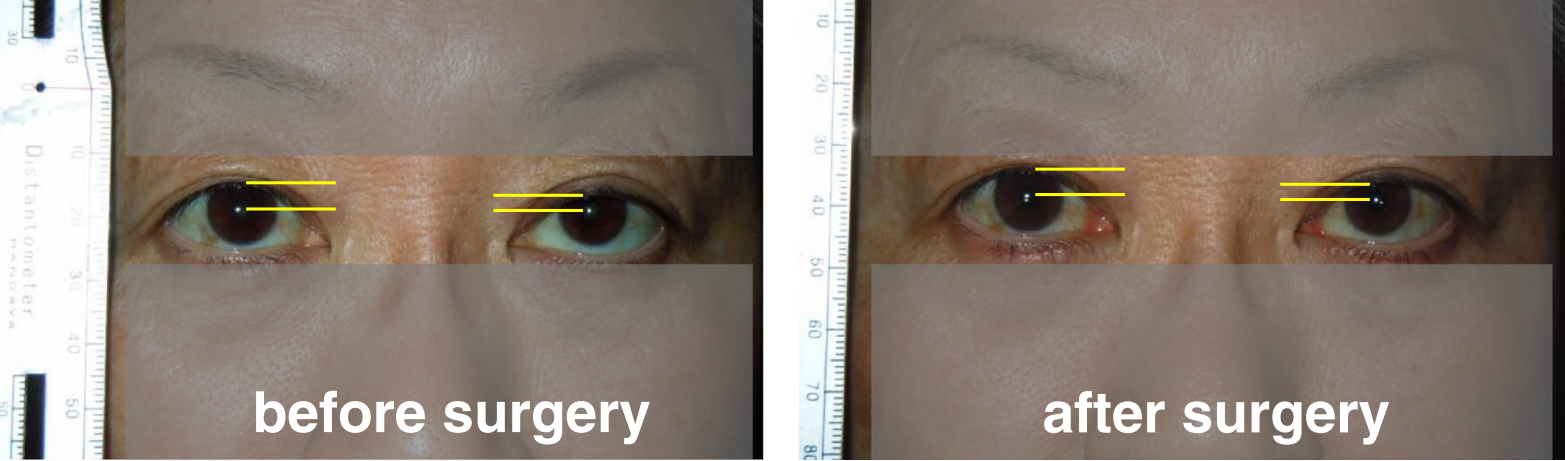
Margin reflex distance1 (MRD1) is the distance between the center of the pupillary light reflex and the upper eyelid margin with the eye in primary gaze (yellow lines)

**Fig. 2:**
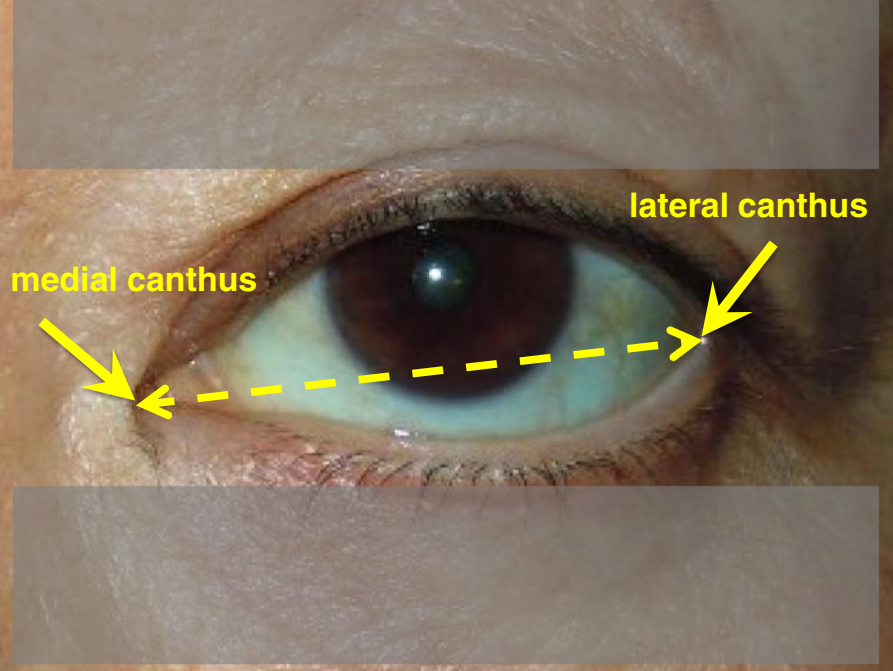
The distance between medial and lateral canthi (broken line) was measured preoperatively

To perform the MRD1 measurements, photographs were taken by a single investigator (N.K.) who had no information about the position of incision for each patient. Photographs were taken preoperatively and at three months postoperatively, and MRD1 was measured using universal ophthalmic measure (Mita PD meter, HE-95, Handaya, Tokyo, Japan) as shown in Fig. 1. All MRD1 measurements were made before mydriatic instillation. We measured the preoperative distance between medial and lateral canthi as shown in Fig. 2. The collected data are available at the LabArchives website (http://www.labarchives.com/bmc).

### Ptosis definition

Ptosis is generally defined as a decrease in the relative position of the upper lid by 2 mm or more compared to the preoperative measurement, which may be the largest difference in MRD1 that can be considered clinically acceptable [9, 10]. Here we used a stricter standard, defining postoperative ptosis as MRD1 difference of ≥0.5 mm between the preoperative and postoperative measurements.

### Statistical analysis

Descriptive statistics are presented as the mean ± standard deviation (SD) or as n (%). A paired *t*-test was used to analyze the change in MRD1 from pre-operation to post-operation measurements. An exact McNemar test was used to compare frequencies in paired binary data. To compare the change in MRD1 (from pre-operation to post-operation) between the temporal incision and the upper incision, we assessed whether the two-sided 95 % confidence interval (CI) for the difference was entirely within the interval −0.5 to 0.5 mm as an equivalence margin. The temporal and upper incisions were considered to be equivalent if the 95 % CI fell entirely within the interval. To explore the risk factors of the postoperative MRD1 reduction, the change in MRD1 was analyzed by using the multivariate regression model including incision position (temporal or upper), preoperative MRD1, and preoperative distance between medial and lateral canthi. A total sample size of 36 patients provided 80 % power that the 95 % CI for the paired mean difference between the two incisions did not exceed ± 0.5 mm, which was the predefined equivalence margin, assuming a mean difference of 0 mm and a SD of 1.0 mm for the change in MRD1. All statistical analyses were performed using SAS 9.4 (SAS institute, Cary, NC, USA). Differences with a *p* value of <0.05 were considered statistically significant.

## Results

A total of 44 patients were initially enrolled. Ten patients could not make it to the hospital on a scheduled day due to personal reasons, and were excluded from this study. Thus, the final analysis included a total of 34 patients: 16 female and 18 male; mean age, 74.7 ± 11.2 years; age range, 69–87 years.

The preoperative MRD1 was 2.27 ± 0.89 mm in the temporal group and 2.24 ± 0.96 mm in the upper group (Table 1). The two incision groups did not significantly differ in pre-operation MRD1 (*p* = 0.764). The postoperative MRD1 was 2.01 ± 1.08 mm in the temporal group versus 2.00 ± 1.05 mm in the upper group (*p* = 0.920). The change in MRD1 from pre-operation to post-operation was −0.26 ± 0.93 mm with the temporal incision and −0.24 ± 0.86 mm with the upper incision. The mean difference in the change in MRD1 between the two incision types was −0.02 mm, with a 95 % CI of −0.24 to 0.20, establishing equivalence between these incisions. We added all data sets used for this study as Additional file 1.

**Table 1:** MRD1 by each incision

MRD1 (mm)	Temporal (*n* = 34)	Upper (*n* = 34)	*p* value^a^	Mean difference (95 % CI)
Pre-operation	2.27 ± 0.89	2.24 ± 0.96	0.764	0.03 (−0.20, 0.27)
Post-operation	2.01 ± 1.08	2.00 ± 1.05	0.920	0.01 (−0.17, 0.30)
Difference between post- and pre-operation values	−0.26 ± 0.93 (95 % CI: −0.58, 0.07) (*p* = 0.115)^b^	−0.24 ± 0.86 (95 % CI: −0.54, 0.06) (*p* = 0.119)^b^	0.849	−0.02 (−0.24, 0.20)
MRD1 decrease of ≥0.5 mm	*n* = 14 (41 %)	*n* = 14 (41 %)	0.392^c^	-

^a^Compared between temporal and upper incisions using a paired *t*-test

^b^Compared between pre- and post-operation values using a paired *t*-test

^c^Compared between temporal and upper incisions using an exact McNemar test

We defined postoperative ptosis as a decrease in MRD1 of ≥0.5 mm from the preoperative measurement to the postoperative measurement. As the mean change from pre-operation to post-operation was −0.26 mm (95 % CI, −0.58 to 0.07) with the temporal incision and −0.24 mm (95 % CI, −0.54 to 0.06) with the upper incision, neither group met this stricter criterion for ptosis. The frequency of postoperative ptosis was 14 (41 %) patients with the temporal incision and 14 (41 %) patients with the upper incision, and did not significantly differ between the two incisions (*p* = 0.392).

The multivariate regression analysis showed that the preoperative MRD1 was significantly associated with the reduction of MRD1 after surgery (*p* = 0.034, Table 2).

**Table 2:** Multivariate regression analysis for the change in MRD1

Factor	Estimate (standard error)	*p* value
Incision position (temporal)	−0.02 (0.11)	0.839
MRD1 in pre-operation (mm)	−0.26 (0.11)	0.034
Distance between medial and lateral canthi in pre-operation (mm)	−0.05 (0.06)	0.382

We examined the distributions of the absolute MRD1 and the change from pre-operation in MRD1. The two measures were basically normally distributed (*p* = 0.07 and *p* = 0.66 by Shapiro-Wilk test, respectively). We added it as Additional file 2.

## Discussion

Our data indicated that postoperative ptosis is rare following phacoemulsification cataract surgery with either an upper or temporal 2.4-mm transconjunctival sclerocorneal incision. Furthermore, there was no significant difference between these two incision types. Interestingly the preoperative MRD1 was significantly associated with the reduction of MRD1 after surgery. Our findings suggest that the decision to perform phacoemulsification cataract surgery with an upper or temporal 2.4-mm transconjunctival sclerocorneal incision may be made based on the surgeon’s personal preference without influencing the risk of postoperative ptosis.

Many factors may be involved in the development of postoperative ptosis. Kaplan et al. suggested that potential causative factors may include local anesthesia, either through a volume effect or myotoxicity; the superior rectus bridle suture; the use of a lid speculum; the size and location of the incision; and upper eyelid edema [1]. They concluded that trauma to the superior rectus muscle by placement of a bridle suture was the most influential factor in postoperative ptosis development. The use of a temporal sutureless incision reduces irritation beneath the upper lid, which is associated with inflammation and edema, and may cause ptosis [12]. With the recently developed technique of phacoemulsification surgery, the incision size and location are likely the most important factors in the development of postoperative ptosis. Our present study focused on the position of the small sutureless incision in phacoemulsification cataract surgery. In their prospective comparative study, Puvanachandra et al. found a postoperative ptosis rate of 18 % in the extracapsular cataract extraction (ECCE) group and 0 % in the phacoemulsification group [9]. They defined ptosis as a decrease in the relative position of the upper lid of 2 mm or more compared to the preoperative measurement, present 6 weeks after surgery. They suggested that the principal factor influencing this difference in ptosis rate was the smaller incision size in the phacoemulsification procedure (10 mm with ECCE compared to 3–4 mm with phacoemulsification).

Kawa et al. compared two groups that underwent phacoemulsification without a bridle suture, but with either peribulbar or retrobulbar anesthesia [13]. Only one patient developed ptosis, and this low rate was attributed to not using a bridle suture. On the other hand, Patel et al. investigated patients undergoing phacoemulsification under peribulbar anesthesia, and found no difference in ptosis rates between those operated using a superior incision and bridle suture and those with a temporal incision with no bridle suture [14]. Ptosis has been also reported after radial keratotomy and laser in situ keratomileusis [15–17], but these procedures are performed under topical anesthesia and with no bridle suture. In the present study, we did not place a bridle suture, and we used a flexible disposable lid speculum in all cases. Proposed mechanisms of ptosis induction due to speculum use include traction on the superior rectus levator complex when a speculum is forced open, and damage to the levator aponeurosis upon contraction of the orbicularis oculi against a rigid speculum. We anticipate that by eliminating as many possible causative factors as possible, we can greatly reduce the risk of postoperative ptosis.

## Conclusions

Our present results indicate that ptosis following phacoemulsification cataract surgery is rare when using either an upper or temporal 2.4-mm transconjunctival sclerocorneal incision, even applying our stricter definition of postoperative ptosis. The longer preoperative MRD1 was significantly associated with the reduction of MRD1 after surgery. These findings suggest that the choice of whether to perform phacoemulsification cataract surgery with an upper or temporal 2.4-mm transconjunctival sclerocorneal incision can be left to the surgeon without concern that either choice will influence ptosis risk. However we should take notice of postoperative ptosis in cases of longer preoperative MRD1.

## Abbreviations

CI, confidence interval; ECCE, extracapsular cataract extraction; MRD, marginal reflex distance; SD, standard deviation

## Additional files

Additional file 1: Preoperative and postoperative MRD1 values, and preoperative distance between medial and lateral canthi. (XLSX 13 kb) Additional file 2: Histograms of the absolute MRD1 and the change from pre-operation in MRD1, We examined the distributions of the absolute MRD1 (left) and the change from pre-operation in MRD1 (right). According to the figure, the two measures were basically normally distributed (*p* = 0.07 and *p* = 0.66 by Shapiro-Wilk test, respectively).
