# Supplementary figures and images for: Influence of upper and temporal transconjunctival sclerocorneal incision on marginal reflex distance after cataract surgery
Source: BMC Ophthalmol. 2016 Jul 7;16:95. doi: 10.1186/s12886-016-0286-1 (PMC4937608; doi:10.1186/s12886-016-0286-1)

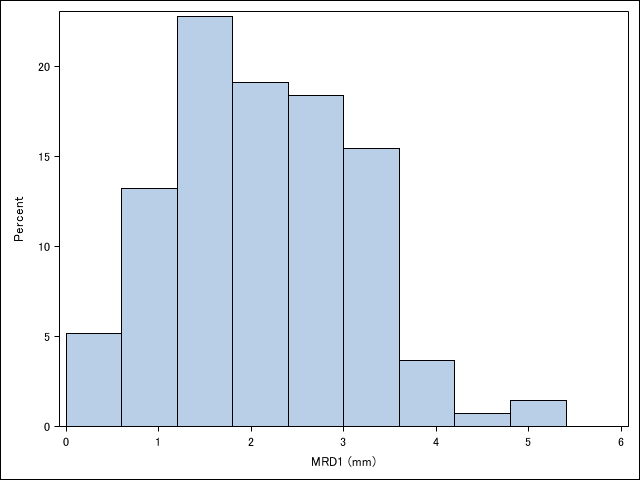

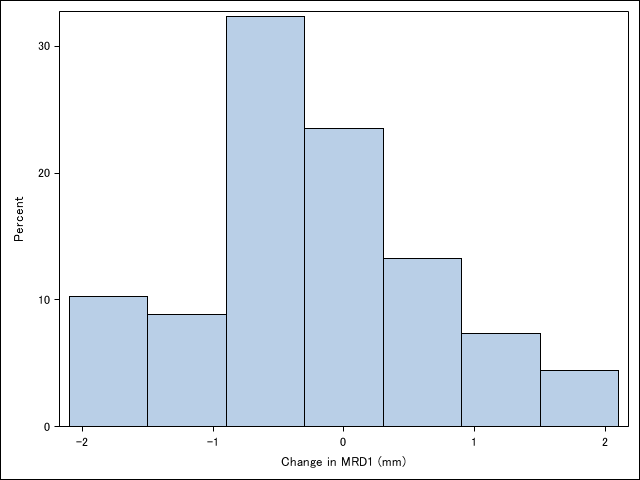

Supplement: Additional file 2: — Histograms of the absolute MRD1 and the change from pre-operation in MRD1, We examined the distributions of the absolute MRD1 (left) and the change from pre-operation in MRD1 (right). According to the figure, the two measures were basically normally distributed (p = 0.07 and p = 0.66 by Shapiro-Wilk test, respectively). [file 12886_2016_286_MOESM2_ESM.docx]
